# Emotional Recognition in Patients With Mesial Temporal Epilepsy Associated With Enlarged Amygdala

**DOI:** 10.3389/fneur.2021.803787

**Published:** 2022-01-21

**Authors:** Giorgi Kuchukhidze, Iris Unterberger, Elisabeth Schmid, Laura Zamarian, Christian Michael Siedentopf, Florian Koppelstaetter, Elke Gizewski, Martin Kronbichler, Gerhard Luef, Hennric Jokeit, Eugen Trinka

**Affiliations:** ^1^Centre for Cognitive Neuroscience, European Reference Network EpiCARE, Department of Neurology, Christian Doppler University Hospital, Paracelsus Medical University of Salzburg, Salzburg, Austria; ^2^Neuroscience Institute, Christian Doppler University Hospital, Salzburg, Austria; ^3^Department of Neurology, Medical University of Innsbruck, Innsbruck, Austria; ^4^Department of Neuroradiology, Medical University of Innsbruck, Innsbruck, Austria; ^5^Department of Radiology, Privatklinik Hochrum, Rum, Austria; ^6^Centre for Cognitive Neuroscience and Department of Psychology, University of Salzburg, Salzburg, Austria; ^7^Swiss Epilepsy Center, Klinik Lengg, Zurich, Switzerland; ^8^Institute of Neuropsychological Diagnostics and Imaging, Karl Landsteiner Institute for Neurorehabilitation and Space Neurology, Salzburg, Austria

**Keywords:** amygdala, temporal lobe epilepsy, epilepsy surgery, fMRI, emotion recognition

## Abstract

**Background:**

Amygdalae play a central role in emotional processing by interconnecting frontal cortex and other brain structures. Unilateral amygdala enlargement (AE) is associated with mesial temporal lobe epilepsy (mTLE). In a relatively large sample of patients with mTLE and AE, we aimed to evaluate functional integration of AE in emotion processing and to determine possible associations between fMRI activation patterns in amygdala and deficits in emotion recognition as assessed by neuropsychological testing.

**Methods:**

Twenty-two patients with drug resistant unilateral mTLE due to ipsilateral AE were prospectively recruited in a large epilepsy unit and compared with 17 healthy control subjects in terms of amygdala volume, fMRI activation patterns and performance in emotion recognition as assessed by comprehensive affect testing system (CATS) and Ekman faces. All patients underwent structural and functional 1.5 Tesla MRI, electro-clinical assessment and neuropsychological testing.

**Results:**

We observed BOLD signal ipsilateral to AE (*n* = 7; group PAT1); contralateral to AE (*n* = 6; group PAT2) and no activation (*n* = 9; group PAT3). In the region of interest (ROI) analysis, beta estimates for fearful face > landscape contrast in the left amygdala region did not differ significantly in patients with left TLE vs. patients with right TLE [*T*_(16)_ = −1.481; *p* = 0.158]. However, beta estimates for fearful face > landscape contrast in the right amygdala region were significantly reduced in patients with right TLE vs. patients with left TLE [*T*_(16)_ = −2,922; *p* = 0.010]. Patients showed significantly lower total scores in CATS and Ekman faces compared to healthy controls.

**Conclusion:**

In our cohort, patients with unilateral mesial TLE and ipsilateral AE, an amygdala could display either functional integration in emotion recognition or dysfunction as demonstrated by fMRI. Perception and recognition of emotions were impaired more in right-sided mTLE as compared to left-sided mTLE. Neuropsychological tests showed deficits in emotion recognition in patients as compared to healthy controls.

## Introduction

The amygdala, a temporal lobe structure is not only of central importance for emotional behavior, but also plays a key role in epileptogenesis and epilepsy (1).

Amygdala enlargement (AE) on MRI without hippocampal structural abnormalities has been associated with a subtype of mesial temporal lobe epilepsy (TLE) (2, 3). On MRI, unilateral sustained AE is characterized by increased size and high signal in T2-weighted images including fluid attenuated inversion-recovery (FLAIR). In so-called non-lesional mesial TLE, amygdala MRI-volumetry may reveal amygdala enlargement ipsilateral to mesial TLE in 12–16% of patients (4, 5). AE may be associated with seizure clusters or even status epilepticus originating from mesial temporal structures. The changes observed on MRI usually vanish in the course of weeks or months correlating with the seizure frequency reduction, returning to either “normal” size or resulting in amygdala atrophy (along with other mesial temporal structures). The sustained AE is usually associated with either autoimmune inflammation, amygdala dysplasia or low-grade tumors (6, 7). However, histopathologically, AE may be associated with unspecific presentation of clustering of hypertrophic neurons and vacuolation with slight gliosis or without gliosis (2).

TLE with AE is a distinct electro-clinical syndrome beginning in middle age adults (8). In patients with AE, electro-clinical presentation of epilepsy may differ from that in patients with pure hippocampal sclerosis (HS). They may have increased tonic components during seizure and generalized ictal EEG onset more frequently than patients with HS (9). Similar to other patients with mesial TLE, patients with AE, particularly those with autoimmune encephalitis, may present with psychosis supposedly due to disrupted circuits in limbic system (10). Increased volume of amygdala was observed in patients with TLE and psychopathology (11). Alterations in Theory of Mind (ToM) abilities in patients with mesial TLE due to HS was associated with disrupted connectivity between amygdala and temporal and frontal brain areas (12). Further, various studies showed impaired emotion processing in patients with TLE due to amygdala lesions (13–17). In a systematic review conducted by Monti and Meletti (18) deficits in fear recognition were observed most often in patients with amygdala lesions, followed by deficits in sadness and disgust recognition. Impaired fear conditioning has also been demonstrated in an animal model of epilepsy with electrical stimulation of amygdala (19).

Similar to other patients with mesial TLE, the patients with AE may develop drug resistant epilepsy requiring epilepsy surgery, rendering ~80% of patients seizure free (20). Anterior temporal lobe resections in such patients usually spare the hippocampus (20). fMRI studies have shown that this type of surgery may be complicated by post-surgical emotional disturbances (21). In general, up to 50% of patients with no psychiatric history may develop symptoms of anxiety and depression shortly after anterior temporal lobe resection (22, 23). Despite this fact, postsurgical emotional disturbances received less attention compared to cognitive changes and the studies were mainly focused on patients with TLE due to hippocampal sclerosis and TLE.

An fMRI paradigm of dynamic fearful faces, which activates amygdala bilaterally in healthy individuals and well-lateralizes mesial TLE, was developed at the Swiss Epilepsy Center (24). In a previous study, we have demonstrated in a small group of patients with possible amygdala dysplasia and mesial TLE that the function might be retained by enlarged amygdala raising concerns about post-surgical deficits (25). In this study, we present a larger sample of patients with unilateral mesial TLE due to ipsilateral amygdala lesion with functional imaging, behavioral and electro-clinical data aiming to evaluate the functional integration of enlarged amygdala in emotion processing and to determine possible associations between fMRI activation patterns in amygdala and deficits in emotion recognition as assessed by neuropsychological testing.

## Methods

### Participants

Twenty-two patients (10 women; mean age 35 years, range 20–57 years) with unilateral AE and ipsilateral mesial TLE were prospectively recruited either at the large out-patient epilepsy unit or in the EEG-video monitoring unit of the Department of Neurology, Medical University of Innsbruck. The inclusion criteria were unilateral non-progressive enlargement of amygdala with ipsilateral drug resistant mesial TLE, normal hippocampi as assessed qualitatively by expert neuro-radiologists as well as by voxel based morphometry. Absence of any other MRI lesions was obligatory.

All patients had drug resistant mesial TLE as determined by electro-clinical presentations. The mean age at seizure onset was 24 years (range 6–50 years); the mean epilepsy duration for the time of the study was 11.9 years (range 1–34 years). All patients underwent video-EEG monitoring with scalp electrodes confirming the origin of seizures from the mesial temporal lobe ipsilateral to AE.

In addition, 17 healthy control subjects with no history of any neurological or psychiatric illness with a mean age of 30 years (range 21–52 years), were matched in age, sex and education to the patient group. The native language of all study participants was German.

### fMRI Task Design

The fMRI paradigm was first developed, validated and applied to healthy subjects and patients with TLE by Schacher et al. (26). Further information related to the selection procedure of the stimuli can be found in Schacher et al. (26). Stimuli were presented in a block design. The paradigm consisted of eight activation and eight baseline blocks each lasting 24 s. The activation condition consisted of 75 brief episodes from thriller and horror films. All episodes showed the faces of actors expressing fear with high intensity. None of the episodes showed violence or aggression. During baseline blocks, 72 short episodes of similar length with landscape video recordings were presented. Video clips of dull domestic landscapes were used owing to their stable low emotional content while their general visual stimulus properties were comparable with the movie clips. Stimuli were presented *via* a back-projection screen, viewed through a tilted overhead mirror. Prior to beginning, subjects were told that they would see presentations of film sequences depicting fearful faces intermixed with landscape film sequences. They were instructed to relax while watching the film and to focus on the eyes of the actors during the activation blocks.

### MRI Acquisition

The MRI-data were obtained using a 1.5 T MR scanner (Siemens Sonata, Erlangen, Germany). MRI sequences included T1-weighted spin echo and gradient echo three-dimensional multiplanar reconstruction images (MPRAGE) ± contrast substance, axial and coronal T2-weighted turbo spin echo, FLAIR and diffusion weighted sequences. Coronal T2 and FLAIR slices were 2–3 mm thick and were acquired at 90° perpendicular to the long axis of hippocampus. There were successive parameters for the anatomic sequence: 176 axial slices with 1-mm single-slice thickness, repetition time (TR) 8.2 ms, echo time (TE) 3.93 ms, 8° flip angle, field of view (FOV) 250 mm, and 288 × 288 matrix.

Functional data were acquired using EPI T2^*^-weighted sequence. The following parameters were applied to measure amygdala activation: 18 coronal slices, 4-mm slice thickness (interslice gap: 0 mm), TR 1,500 ms, TE 35 ms, 75° flip angle, FOV 220 mm, matrix size 64 × 64 (voxel size 2.75 × 2.75 × 4 mm), reconstructed into an image matrix of 128 × 128. Coronal slices were geared orthogonally to the hippocampal formation and were spread over the anterior temporal lobe.

### Criteria for the Assessment of Amygdala Structural Abnormalities

Amygdala lesion was determined qualitatively by visual assessment by expert neuroradiologists and quantitatively by voxel based volumetry. All patients had unilaterally enlarged amygdala as seen on T1- and T2-weighted images without uptake of contrast substance and had an increased signal in the FLAIR sequence. All patients underwent at least two MRIs with an interval of at least 6 months. The MRI changes were stable in consecutive MRIs suggesting a non-progressive abnormality. Hippocampal volumetry showed normal hippocampi in all patients.

### Single Subject Analysis of fMRI Data

Image analysis for revealing significant brain activation based on changes in blood oxygen level dependant (BOLD) signal was performed on each subject's fMRI data by using statistical parametric mapping (SPM12, Wellcome Department of Cognitive Neurology, London, UK; http://www.fil.ion.ucl.ac.uk/spm/software/spm12/) under MATLAB R2013a (MathWorks Inc., Natick, MA, USA). The functional data sets of each patient were motion corrected after discarding the first three volumes to allow signal stabilization. Eventually 269 volumes per series were utilized for data analysis. Anatomical high-resolution images were co-registered to a mean functional image of each subject. Images were normalized spatially and smoothed using an 8 mm FWHM Gaussian kernel. A statistical analysis on the basis of the general linear model (GLM) was conducted as implemented in SPM12. The delta-function of the block onsets was convolved with the canonical form of the hemodynamic response function for a duration corresponding to the block length, to generate the model time courses for the two conditions of the paradigm. A high-pass filter (1/288 Hz) was used to remove low frequency drifts. SPM maps of the contrast of voxels with increased intensity during “active” blocks (fearful faces) in relation to the contrast block (landscapes) in the whole brain were computed. Clusters of activation were reported as significant, when they surpassed an initial threshold of *p* < 0.001 (uncorrected) and had a FEW (family-wise error) corrected *p*-value of *p* < 0.05 on cluster level.

### Group Analysis of fMRI Data

For preprocessing and statistical analysis within group comparison, SPM12 software (http://www.fil.ion.ucl.ac.uk/spm/12/), running in a MATLAB R2013a environment (Mathworks Inc., Natick MA, USA), and additional functions from AFNI (Analysis of Functional Neuro-Images, https://afni.nimh.nih.gov/) were used. Functional images were realigned, de-spiked (with the AFNI 3D despike function) unwarped and corrected for geometric distortions using the fieldmap of each participant and slice time corrected. The high resolution structural T1-weighted image of each participant was processed and normalized with the CAT12 toolbox (Computational Anatomy Toolbox 12, http://dbm.neuro.uni-jena.de/cat) using default settings. Each structural image was segmented into gray matter (GM), white matter (WM) and cerebro-spinal fluid (CSF) and denoised. Then each image was warped into Montreal Neurological Institute (MNI) space by registering it to the DARTEL template provided by the CAT12 toolbox *via* the high-dimensional DARTEL (diffeomorphic anatomical registration through exponentiated Lie algebra) (27) registration algorithm. Based on these steps, a skull stripped version of each image in native space was created.

To normalize functional images into MNI space, the functional images were co-registered to the skull stripped structural image and the parameters from the DARTEL registration (27) were used to warp the functional images, which were re-sampled to 3 × 3 × 3 mm voxels and smoothed with a 6 mm FWHM Gaussian kernel.

Statistical analysis was performed with a GLM two staged mixed effects model. In the subject-specific first level model, each condition was modeled by convolving stick functions at its onsets with SPM12's canonical hemodynamic response function (target trials and start and end messages were modeled as separate events of no interest, the model also included the six motion parameters and six noise regressors, reflecting physiological noise components obtained from FIACH (Functional Image Artifact Correction Heuristic) (28) as regressors of no interest. Parameter estimates for each condition were calculated *via* these first level GLM, using a temporal high-pass filter (cutoff 128 s) to remove low-frequency drifts and modeling temporal autocorrelation across scans with a first-order autoregressive process (AR) (29).

For voxel-based group analyses, contrast images (fearful faces > landscapes) for effects of interest were calculated at the first level and used for second level analyses using one-way ANOVAS with patients groups and healthy controls. A threshold of *p* = 0.005 (uncorrected) with a cluster-level FDR threshold of *p* = 0.05 were used.

### Region of Interest (ROI) Analyses

In a first step, the Regions of interest (ROIs) were defined by manual segmentation of the amygdalae by 3D-Slicer (htpps://www.slicer.org/, Pieper, Lorensen, Schroeder and Kikinis) (30) for patients and healthy controls. In a second step, we extracted the beta estimates of these individual ROIs for the first-level contrast (fearful faces > landscapes) which were further used for group analyses. Peak coordinates and voxel extent are reported in the Supplementary Table 1. Subsequent analyses were done using IBM SPSS Statistics 20®.

### Amygdala Volumetry

T1-weighted volume datasets were normalized to Montreal Neurological Institute template space using DARTEL (27) and segmented into different brain compartments—GM, WM, and CSF using the unified segmentation algorithm of SPM12 with default parameters. Volumetric measures of brain structures were calculated by voxel-by-voxel multiplication and subsequent integration of normalized and modulated component images (GM, WM, or CSF) with predefined masks in the same space. Masks for amygdala were derived from the Harvard-Oxford atlas of subcortical structures distributed with the Oxford Center for Functional MRI of the Brain Software Library (FSL) package. Amygdala volumes were controlled for age, sex, and total cerebral volume (31–34). GM, WM, and CSF volumes and intracranial volume (ICV) were determined by the “tissue volumes” utility of SPM12 (35).

### Neuropsychological Tests

Thirty-tree participants (18 patients and 15 healthy controls) underwent neuropsychological testing including the multiple-choice vocabulary test (MWT-B) which served as estimate of crystalline verbal intelligence (36). Further, the test battery comprised tests for emotion recognition and a self-report questionnaire for depression and anxiety symptoms (see below).

#### Emotion Recognition

##### Comprehensive Affect Testing System (CATS)

The Comprehensive affect testing system (CATS) (37) is a computerized measure of emotion processing previously used in studies with epilepsy patients (12, 38). It aims to assess the perception of facial expressions, prosody, and linguistically presented emotional material and therefore employs visual and auditory modalities of communication (37). Instructions as well as verbal and auditory stimuli for CATS tasks were translated into German at the Swiss Epilepsy Center.

The CATS consists of 13 subtests: 11 emotion tasks and two control tasks assessing facial identification, emotion matching with and without verbal denotation (e.g., in some tasks both emotional faces and the name of the target emotion is displayed on the screen, whereas in other tasks no additional verbal cues are given), emotional tone or prosodic processing with and without verbal denotation (e.g., in some tasks both emotional prosody and the name of the target emotion is displayed on the screen, whereas in other tasks no additional verbal cues are given), and with conflicting or congruent semantic content. Emotional stimuli covered happy, sad, angry, surprised, disgusted, fearful, or neutral mood.

**Composite scales**. Each item within a subtest is scored as either correct or incorrect, and items are summed to obtain a raw score for each of the 13 subtests. Data from the 11 emotion related subtests were combined and reduced to five different composite scales: Simple Facial Scale (Subtests 2 and 5), Complex Facial Scale (Subtests 7, 8, 13), Prosody Scale (Subtests 4, 6, 9), Lexical Scale (Subtests 10), and Cross-Modal Scale (Subtest 11 and 12).

**Quotient scales**. Broader scales, based on mode of communication (facial affect and prosody) and emotion *per se*, are also generated. The Affect Recognition Quotient is obtained by combining the two facial scales; the Prosody Recognition Quotient is identical to the Prosody Scale, and the Emotion Recognition Quotient is an overarching scale and includes all 11 emotional subtests.

**Discrete emotion scales**. There are additional scales for each of the six basic emotions that provide information about performance based on type of emotion. Items in the discrete emotion scales test facial affect recognition and are taken from Subtests 5, 7, 8, and 13.

##### Ekman Faces

The Ekman 60 Faces Test is a well-known neuropsychological tool assessing emotion recognition from facial expressions of basic emotions. It consists of photographs from the Ekman and Friesen series of Pictures of Facial Affect (39), which has been the most widely used and validated series of photographs in facial expression research. The faces of 10 actors (six female, four male) were shown, each displaying the six basic emotions (happiness, sadness, disgust, fear, surprise, and anger). The maximum test score indicating best performance is 60 for all six emotions and 10 for each basic emotion. The computer software for the test was available on CD-ROM. Patients were allowed unlimited time for the response.

##### Anxiety and Depression Scale

The HADS measures levels of anxiety and depression during the last week. It consists of an anxiety subscale (HADS-A) and a depression subscale (HADS-D), each containing seven items scoring from 0 to 3 (40). Total scores in each subscale range from zero to 21, with higher scores indicating pronounced anxiety- and depression-related symptoms. We classified scores below eight as normal, between eight and 10 as mild, and above 10 as clinically significant (40).

### Statistics

Analyses were carried out using SPSS Version 20. Two-by-two comparisons were performed by means of Mann–Whitney-*U*-Test for non-parametric independent samples and unpaired *t*-test for parametric samples. Comparisons of more than two groups were performed by means of the Kruskal–Wallis test or one-way ANOVA.

Significance was set at α <0.05 and all *p*-values were adjusted for multiple comparison using the Benjamini-Hochberg FDR correction method. Correlation of independent as well as dependent variables with each other and with variables such as behavioral data were tested using the Spearman's rank correlation coefficient.

### Ethics Statement

The study was performed according to the Declaration of Helsinki and approved by the local ethics committee of the Medical University of Innsbruck. Written informed consent was obtained from all participants prior to study tests.

## Results

An overview of demographic data of patients and healthy controls is shown in Table 1. A significant difference between patients and healthy controls was seen in the estimated verbal IQ, with healthy controls achieving higher scores.

**Table 1:** Characteristics of patients and healthy controls.

**Variable**	**Healthy controls (*n* = 17)**	**TLE patients (*n* = 22)**	**Mann-Whitney-*U*-Test / Chi^**2**^-Test/(Fisher's exact test**
Age in years, mean (SD)	31 (8.6)	35 (10.3)	*p* = 0.193
Sex (female; male)	9; 8	10; 12	*p* = 0.643
Education in years, mean (SD)	10.73 (5.7)	12.14 (3.5)	*p* = 0.262
Handedness (right; left)	15; 2	19; 3	*p* = 0.862
MWT-B (estimated IQ), mean (SD)	115.40 (12.4)	101.82 (10.35)	*p* = 0.008

### fMRI Single Subject Analysis

**Figure 1:**
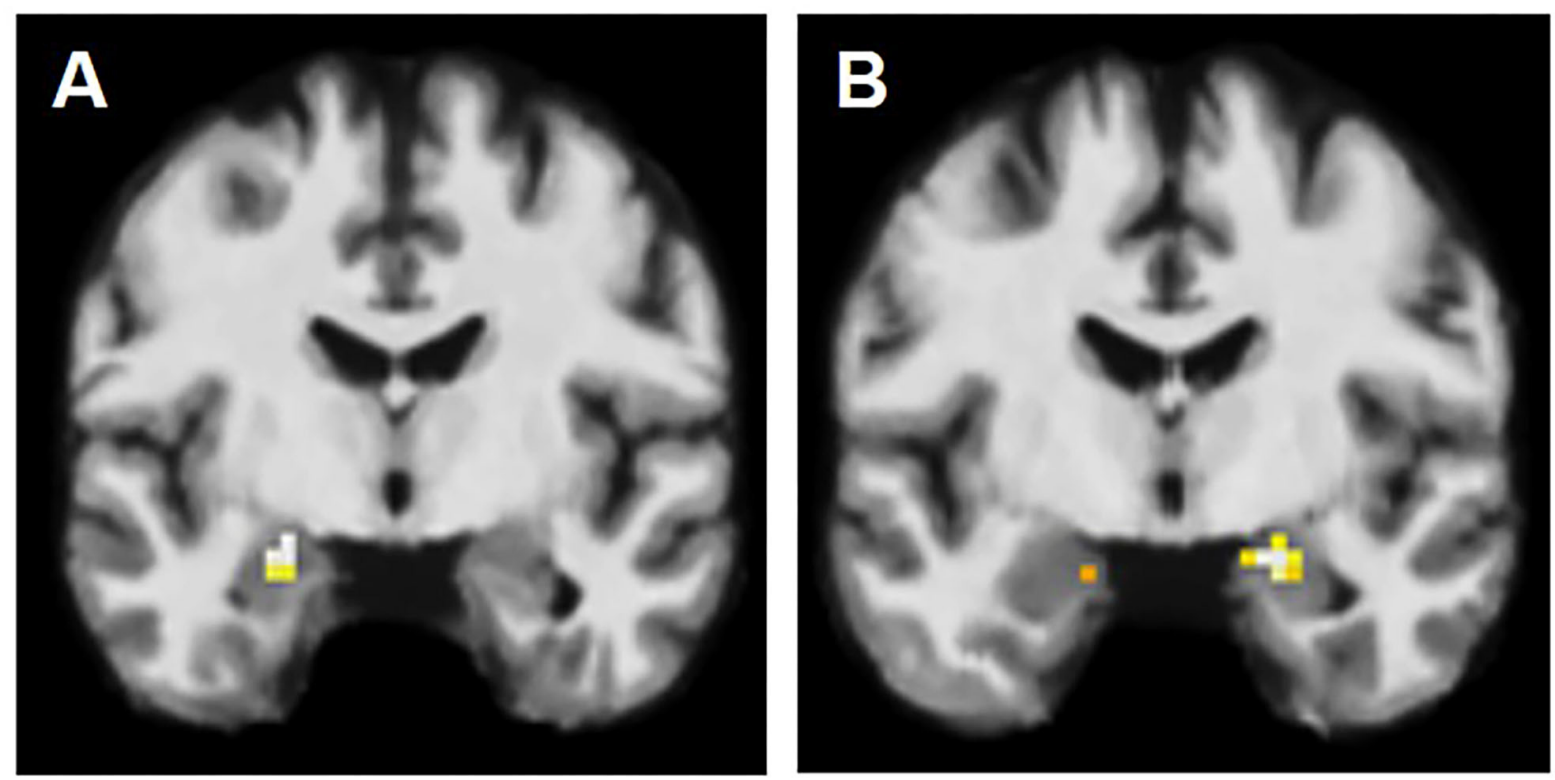
fMRI activation patterns in amygdala lesions. **(A)** BOLD signal in left amygdala, contralateral to the AE on the right. **(B)** BOLD signal in amygdala bilaterally, however more on the right than on the left (left-sided AE).

Blood oxygenation dependent (BOLD) signal was elicited in 13 (59%) patients and 12 (71%) healthy controls. The BOLD signal was seen most frequently bilaterally in amygdalae in both groups (Figure 1). However, in healthy controls it was more common compared to the patients (47 vs. 23% respectively), without reaching a statistically significant difference.

In patients, we observed the following activation patterns in fMRI task in relation to AE: seven patients had ipsilateral activation (group PAT1), suggesting therefore functional integration of enlarged amygdala. In six patients, the BOLD signal was observed solely in the amygdala contralateral to the AE (group PAT2) (Figure 1) and in nine patients, there was no BOLD signal registered in either amygdala (group PAT3), suggesting dysfunction of amygdala in these two groups (Table 2).

**Table 2:** BOLD signal in patients.

Ipsilateral to amygdala lesion	7 (7 TLE L)
Contralateral to amygdala lesion	6 (4 TLE L; 2 TLE R)
No BOLD	9 (6 TLE L; 3 TLE R)

We did not analyze the presence of BOLD signal in relation to the AE side due to small sample size in each sub-group (the data presented in Tables 2, 3).

**Table 3:** fMRI activation patterns in TLE patients and healthy controls.

	**No BOLD signal**	**BOLD signal bilaterally**	**BOLD signal on the left**	**BOLD signal on the right**	**Total**
Healthy controls (N, %)	5 (29%)	8 (47%)	2 (12%)	2 (12%)	17 (100%)
Patients (N, %)	9 (41%)	5 (23%)	4 (18%)	4 (18%)	22 (100%)
TLE L (N)	6	5	**2 (ipsilateral)**	**4 (contralateral)**	17
TLE R (N)	3	0	**2 (contralateral)**	0	5

### Amygdala Volumetry

Amygdala volumes in 17 healthy controls were normally distributed. The mean total volume of amygdala in healthy controls was 3.6 (SD 0.2) cm^3^. The right and left amygdala volumes were not statistically different, however slightly larger on the right with a mean volume of 1.9 (SD 0.1) cm^3^ and left amygdala with a mean volume of 1.7 (SD 0.1) cm^3^. The left amygdala was significantly larger in patients with left mesial TLE compared to the left amygdala in healthy controls: mean volume 1.8 cm^3^ (SD 0.1) vs. 1.7 cm^3^ (SD 0.1), respectively; Student's *t*-test *p* = 0.015, 2-tailed. The mean volume of right amygdala was higher in patients with right mesial TLE compared to the volume of the right amygdala in the healthy controls, without reaching statistically significant difference: mean 2.0 cm^3^ (SD 0.3) vs. 1.9 (SD 0.1) cm^3^, respectively; Student's *t*-test *p* = 0.124, 2-tailed.

### fMRI Group Comparisons

#### Whole Brain Analysis

There was no significant difference in activation patterns when the whole group of patients was compared with healthy controls. Sub-group analyses regarding patients with left mesial TLE vs. healthy controls as well as patients with right mesial TLE vs. healthy controls also did not show any significant difference [all *p* > 0.005 (uncorrected)].

#### ROI Analyses

Beta estimates were extracted from right and left amygdalae, as defined by manual segmentation.

Beta estimates for fearful face > landscape did not differ significantly within the patient groups (PAT1, PAT2, and PAT3). Beta estimates for fearful face > landscape contrast in the left amygdala region did not differ significantly in patients with left TLE vs. patients with right TLE [*T*_(16)_ = −1.481; *p* = 0.158]. However, beta estimates for fearful face > landscape contrast in the right amygdala region were significantly reduced in patients with right TLE vs. patients with left TLE [*T*_(16)_ = −2,922; *p* = 0.010]. No significant difference was found when comparing beta-estimates of the unilaterally enlarged amygdala to the contralateral amygdala within the patient group [*T*_(16)_ = −1.433, *p* = 0.170]. The results of the individual ROIs are presented in the Supplementary Table 1.

#### Behavioral Tests

Neuropsychological data were not carried out in four patients and two healthy controls. Therefore, those subjects could not be included in further analysis regarding neuropsychological tests on emotion recognition.

##### Comprehensive Affect Testing System (CATS)^*^

**Quotient scales:** Comparison of all patients with healthy controls showed significantly worse performance in the Emotion Recognition Quotient. In the Affect Recognition Quotient and the Prosody Recognition Quotient, no difference was observed between the two groups. For results, see Table 4.

**Table 4:** Performance of TLE patients and healthy controls on the CATS quotients expressed as *Z* scores.

**Variable**	**Healthy controls (*n* = 15) Med (min-max)**	**TLE patients (*n* = 18) Med (min-max)**	**Mann-Whitney-*U*-Test**
Affect Recognition Quotient	1.00 (-1.00 – 2.00)	0.20 (-1.3 – 1.00)	*p* = 0.106
Prosody Recognition Quotient	−0.40 (-2.90 – 2.00)	−1.00 (-4.70 – 0.80)	*p* = 0.329
Emotion Recognition Quotient	0.40 (-1.60 – 2.20)	−0.85 (-3.50 – 0.40)	*p* = 0.023^*^
Simple Facial Scale	0.40 (-1.60 – 2.20)	0.60 (-0.90 – 1.20)	*p* = 0.829
Complex Facial Scale	0.90 (-1.30 – 2.00)	−0.20 (-2.00 – 1.10)	*p* = 0.046^*^
Prosody Scale	−0.40 (-0.50 – 1.60)	−1.00 (-4.60 – 0.80)	*p* = 0.329
Lexical Scale	−0.80 (-1.30 – 0.30)	−0.90 (-2.70 – 0.30)	*p* = 0.011^*^
Discrete Emotion Scales:			
Happy	0.30 (0.30 – 0.30)	0.30 (-2,60 – 0.30)	*p* = 0.744
Surprised	0.10 (-1.70 – 1.00)	−0.80 (-2.70 – 1.00)	*p* = 0.422
Fear	1.10 (-2.30 – 1.90)	0.20 (-2.30 – 1.50)	*p* = 0.214
Sad	−0.40 (-2.50 – 0.30)	−1.50 (-6.60 – 0.30)	*p* = 0.829
Angry	0.00 (-1.00 – 1.60)	−1.00 (-1.50 – 1.10)	*p* = 0.035^*^
Disgusted	0.70 (-2.00 – 1.20)	−0.15 (-2.00 – 1.00)	*p* = 0.488

* *Mann-Whitney-U-Test is significant at the p <0.05 level (two-tailed)*.

When comparing the three patient groups (PAT1, PAT2, and PAT3), no significant difference was found in the three quotient scales (all *p* > 0.05).

**Composite scales:** Comparison of all patients with the healthy control group showed significant differences in the Complex Facial Scale and in the Lexical Scale. Patients performed significantly worse than healthy controls. The remaining two Composite Scales (Simple Facial Scale, Prosody Scale) did not show significant differences (all *p* > 0.05). When comparing the three patient groups (PAT1, PAT2, and PAT3), no significant difference was found in all four composite scales (all *p* > 0.05).

**Discrete emotion scales:** Patients performed significantly worse compared to healthy controls in recognition of anger. In all other Discrete emotion scales, no significant differences were observed. Furthermore, no differences were observed when the three groups of patients (PAT1, PAT2, and PAT3) were compared (all *p* > 0.05).

##### Ekman 60 Faces Test^*^

The patient group performed significantly worse compared to healthy controls in emotion recognition as assessed by the Ekman 60 Faces Test (sum of all six sub-scales) (Table 5). However, no significant difference can be found in sub-scales, each presenting one of the six basic emotions (all *p* > 0.05).

**Table 5:** Results of each group on the Ekman 60 Faces Test.

**Ekman-Scales**	**Healthy controls (*n* = 15) sum correct answers Med (min-max)**	**TLE patients (*n* = 18) sum correct answers Med (min-max)**	**Mann-Whitney-*U*-Test**
Anger	9 (6–10)	8 (5–10)	*p* = 0.329
Disgust	9 (5–10)	7,5 (3–10)	*p* = 0.102
Fear	6 (2–10)	4.5 (1–10)	*p* = 0.102
Happiness	10 (9–10)	10 (8–10)	*p* = 0.997
Sadness	8 (7–10)	8 (2–10)	*p* = 0.898
Surprise	9 (7–10)	9 (5–10)	*p* = 0.931
Total score	53 (44–57)	44.5 (38–59)	*p* = 0.017^*^

* *Mann-Whitney-U-Test is significant at the p <0.05 level (two-tailed)*.

When comparing the three patient groups (PAT1, PAT2, and PAT3), no significant difference could be found (all *p* > 0.05).

^*^The influence of verbal IQ as a potential confounding factor was assessed by explorative univariate ANOVA for the CATS and Ekman faces measures where significant group differences were detectable by means of Mann-Whitney *U*-tests (i.e., Emotion Recognition Quotient, Complex Facial Scale, Lexical Scale, Angry, Ekman total score). Significance was set at a α <0.05 one-tailed. The group difference in the Lexical Scale was the only one that did not reach significance after controlling for verbal IQ.

##### Hospital Anxiety and Depression Scale (HADS)

In both anxiety and depression subscales, the majority of patients and healthy controls scored in the normal range. Very few participants in both groups scored above seven, indicating mild to clinically significant anxiety- or depression-related disorders. Chi2-tests indicated that the distribution of normally scoring participants vs. mild-to-clinically relevant disorders did not differ between the two groups (both *p* > 0.1) (Table 6).

**Table 6:** Results of each group on HADS.

	**Healthy controls**	**TLE patients**	**Chi2-test (Fisher's exact test)**
Anxiety (HADS-A)			0.458
Score ≤ 7	12 (80%)	13 (65%)	
Score 8–10	3 (20%)	5 (25%)	
Score ≥11	0 (0%)	2 (10%)	
Depression (HADS-D)			0.244
Score ≤ 7	15 (100%)	17 (85%)	
Score 8–10	0 (0%)	3 (15%)	
Score ≥11	0 (0%)	0 (0%)	

##### Correlations

Ekmann total score correlated with the CATS total score (sum of all 13 subtests) (*r* = 0.74, *p* < 0.001). Both total scores correlated significantly with verbal IQ (Ekman: *r* = 0.60, *p* < 0.001; CATS: *r* = 0.62, *p* < 0.001).

Regarding depression and anxiety symptoms, both scores correlated with the Ekmann total score (*r* = −0.487, *r* < 0.05 / *r* = −0.438, *r* < 0.05), as well as the CATS total score (*r* = −598, *p* < 0.001 / *r* = −585, *p* < 0.001).

## Discussion

In this prospective study on 22 patients with unilateral drug resistant mTLE due to ipsilateral AE, we have demonstrated that enlarged amygdala might display both, functional integration in emotion processing as well as dysfunction, as assessed by “fearful faces” fMRI paradigm. Enlarged amygdala on the right was dysfunctional in patients with right mTLE compared to those with left mTLE. The patients performed worse compared to healthy controls in emotion recognition as shown by the CATS and Ekman faces.

Enlargement of amygdala is a rare condition which may be associated with mTLE (3). The changes observed on MRI might be caused by different etiologies such as autoimmune limbic encephalitis, dysplasia or low-grade tumor. Autoimmune limbic encephalitis is the most frequent cause of lesional mTLE in adults (41). Aside from epileptic seizures, which poorly respond to drugs, patients may present with acute psychosis (41, 42). On MRI, swelling of mesial temporal structures with high signal in T2-weighted sequences and uptake of contrast substance are observed in an acute phase (41, 43). These changes may be uni-or bilateral, they either resume completely within few months or result in a volume reduction of amygdala and hippocampus, causing mesial temporal lobe sclerosis (43, 44). In a serum or cerebro-spinal fluid (CSF) of patients with limbic encephalitis, either onconeural (mainly Hu and Ma2 antibodies) or various non-paraneoplastic antibodies (e.g., VGKC, NMDA-R) may be detected (41, 42).

Amygdala is considered dysplastic if the patients do not have any clinical or laboratory evidence of limbic encephalitis and the MRI changes remain stable over a long period of time (41). Low grade (e.g., astrocytoma WHO grade II) or dysplastic tumors (e.g., developmental neuroepithelial tumor or ganglioglioma) are diagnosed on histopathology in about 15% of patients with AE (42).

In our cohort of patients, we could not determine the underlying etiology as the histology was not available in a pre-surgical setting and the majority of patients were not tested for serum or CSF antibodies. All our patients underwent at least two serial MRIs with an interval of at least 6 months and no lesion progression or contrast substance uptake was demonstrated. Therefore, we presume that the patients in our series did not have a high-grade tumor affecting amygdala. The heterogeneity of fMRI response in patients might be due to different etiology of AE. We could speculate that those with amygdala dysplasia might demonstrate dysfunction as opposed to acquired lesions such as autoimmune limbic encephalitis or low-grade tumor, which could maintain function. Congenital lesions such as malformations of cortical development due to abnormal neuronal proliferation (focal cortical dysplasia type II, tuberous sclerosis, etc.) usually do not bear function as demonstrated by fMRI studies (45, 46) and invasive EEG recordings (47). Other possible reasons for amygdala dysfunction might be epilepsy features irrespective of its etiology, e.g., duration of epilepsy, seizure frequency, seizure types etc. However, in the regression analysis, we could not find any association between different epilepsy features and fMRI activation patterns.

The side of temporal lobe damage might play a critical role in perception of fear as left- and right-sided TLE patients displayed different fMRI activation patterns in our series. In patients with right TLE, amygdala displayed either no BOLD signal or activation on the contralateral side; whereas patients with left-sided TLE showed activation of damaged amygdala in 41% of cases. Right amygdala has been implicated in the literature as a key structure for fear processing and that its function cannot be fully compensated in case of right-sided mesio-temporal lobe damage (48).

In a systematic review, it has been shown that facial emotion recognition of all six basic emotions (anger, disgust, fear, sadness, happiness, and surprise) was impaired in patients with TLE compared to healthy controls with the largest effect size for fear (49). Interestingly, poorer ability of facial emotion recognition was observed in patients with right-sided TLE (18), which is in line with our findings as we demonstrated dysfunction of right enlarged amygdala in patients with ipsilateral mTLE. Similar to our findings, Toller et al. demonstrated by dynamic fearful faces fMRI that right mTLE was associated with reduced activity in the right amygdala, suggesting its mediating role in the emotional awareness of empathic experiences of fear (50). In patients with mTLE, the deficits in facial emotion perception are contributed to the disruptions of functional networks, which are more pronounced in right mTLE (51). Children and adolescents with mTLE also demonstrate deficits in perception of different emotions, especially of fear (52). These deficits were observed in patients with both left and right mTLE, however, the deficits were more prominent in the latter (52). Earlier onset of seizures was associated with poorer recognition of facial expression of emotions (52). The earlier the damage to the right mesial temporal structure, the greater the deficit in fear recognition and in fMRI activation magnitude in patients with right-sided TLE (53). In our series, we did not observe any association between the duration of epilepsy and the fMRI activation patterns. However, the neurobiology of the lesion and the time of the insult could determine dysfunction of the right amygdala.

Amygdala abnormalities contribute to comorbid affective disorders in TLE. Interictal anxiety and depression are common in patients with pharmacoresistant TLE due to amygdala damage. At the same time, patients with major depression show amygdala dysfunction in fMRI. The fMRI reactivity of amygdala to emotion facial impressions is associated with greater chances of symptom improvement in patients with major depression (54). In patients with drug resistant mTLE, a pre-operative assumption of the post-surgical risks of developing emotional disturbances is of great importance. Bonneli et al. have shown that the greater the pre-operative fMRI activation in right amygdala, the higher the chance of developing post-operative depression and anxiety (21). This was not true, however, for patients with left-sided TLE. As our study was pre-surgical, we cannot judge the deficits following epilepsy surgery.

The main limitations of this study are related to a small number of patients in analyzed subgroups and the pre-surgical nature of the study, which precludes the histological diagnosis of AE. Furthermore, the vast majority of patients of this series were not tested for autoimmune antibodies, which would potentially contribute to the diagnosis of limbic encephalitis. Therefore, in the absence of histological and immunological data, we could not judge upon the neurobiology of dysfunctional or functionally integrated enlarged amygdala.

## Conclusion

In this study, we could show that in patients with unilateral mesial TLE and ipsilateral AE, an amygdala could display either functional integration in emotion recognition or dysfunction as demonstrated by fMRI. The results of neuropsychological tests display deficits in emotion recognition in patients as compared to healthy controls. These results are in line with the current evidence and support the notion that perception and recognition of emotions are commonly impaired in mTLE, more in right-sided mTLE as compared to left-sided mTLE.

## Data Availability Statement

The original contributions presented in the study are included in the article/Supplementary Material, further inquiries can be directed to the corresponding author/s.

## Ethics Statement

The studies involving human participants were reviewed and approved by Ethics Committee of the Medical University of Innsbruck, Austria. The patients/participants provided their written informed consent to participate in this study. Written informed consent was obtained from the individual(s) for the publication of any potentially identifiable images or data included in this article.

## Author Contributions

GK and IU contributed significantly to conception and design of the presented paper, acquisition, analysis, and interpretation of the data as well as drafting of the paper. ES, LZ, CS, FK, and MK contributed to acquisition and analysis of data and revising the paper for intellectual content. GL, EG, HJ, and ET contributed significantly to conception of the study, interpretation of the results, and gave final approval of the submitted version of the manuscript. All authors contributed to the article and approved the submitted version.

## Funding

This study was supported by FWF, Austrian Science Fund (Project Number P21636-B18). This work was generated within the European Reference Network for Rare and Complex Diseases EpiCARE.

## Conflict of Interest

The authors declare that the research was conducted in the absence of any commercial or financial relationships that could be construed as a potential conflict of interest.

## Publisher's Note

All claims expressed in this article are solely those of the authors and do not necessarily represent those of their affiliated organizations, or those of the publisher, the editors and the reviewers. Any product that may be evaluated in this article, or claim that may be made by its manufacturer, is not guaranteed or endorsed by the publisher.

## References

[1] Aroniadou-Anderjaska V Fritsch B Qashu F Braga MF. Pathology and pathophysiology of the amygdala in epileptogenesis and epilepsy. Epilepsy Res. (2008) 78:102–16. 10.1016/j.eplepsyres.2007.11.011 18226499 PMC2272535

[2] Minami N Morino M Uda T Komori T Nakata Y Arai N. Surgery for amygdala enlargement with mesial temporal lobe epilepsy: pathological findings and seizure outcome. J Neurol Neurosurg Psychiatry. (2015) 86:887–94. 10.1136/jnnp-2014-308383 25224675

[3] Mitsueda-Ono T Ikeda A Inouchi M Takaya S Matsumoto R Hanakawa T. Amygdalar enlargement in patients with temporal lobe epilepsy. J Neurol Neurosurg Psychiatry. (2011) 82:652–7. 10.1136/jnnp.2010.206342 21047879

[4] Coan AC Morita ME de Campos BM Yasuda CL Cendes F. Amygdala enlargement in patients with mesial temporal lobe epilepsy without hippocampal sclerosis. Front Neurol. (2013) 4:166. 10.3389/fneur.2013.00166 24298266 PMC3829468

[5] Singh P Kaur R Saggar K Singh G Aggarwal S. Amygdala volumetry in patients with temporal lobe epilepsy and normal magnetic resonance imaging. Pol J Radiol. (2016) 81:212–8. 10.12659/PJR.896077 27231493 PMC4865273

[6] Beh SMJ Cook MJ D'Souza WJ. Isolated amygdala enlargement in temporal lobe epilepsy: a systematic review. Epilepsy Behav. (2016) 60:33–41. 10.1016/j.yebeh.2016.04.015 27176882

[7] Kim DW Lee SK Chung CK Koh YC Choe G Lim SD. Clinical features and pathological characteristics of amygdala enlargement in mesial temporal lobe epilepsy. J Clin Neurosci. (2012) 19:509–12. 10.1016/j.jocn.2011.05.042 22321366

[8] Malter MP Widman G Galldiks N Stoecker W Helmstaedter C Elger CE. Suspected new-onset autoimmune temporal lobe epilepsy with amygdala enlargement. Epilepsia. (2016) 57:1485–94. 10.1111/epi.13471 27418040

[9] Du X Usui N Terada K Baba K Matsuda K Tottori T. Semiological and electroencephalographic features of epilepsy with amygdalar lesion. Epilepsy Res. (2015) 111:45–53. 10.1016/j.eplepsyres.2015.01.007 25769372

[10] Kandratavicius L Ruggiero RN Hallak JE Garcia-Cairasco N Leite JP. Pathophysiology of mood disorders in temporal lobe epilepsy. Revista brasileira de psiquiatria. (2012) 34(Suppl.2):S233–45. 10.1016/j.rbp.2012.08.003 23429849

[11] Trimble MR Van Elst LT. The amygdala and psychopathology studies in epilepsy. Ann N Y Acad Sci. (2003) 985:461–8. 10.1111/j.1749-6632.2003.tb07100.x 12724177

[12] Broicher SD Frings L Huppertz HJ Grunwald T Kurthen M Kramer G. Alterations in functional connectivity of the amygdala in unilateral mesial temporal lobe epilepsy. J Neurol. (2012) 259:2546–54. 10.1007/s00415-012-6533-3 22688567

[13] Benuzzi F Meletti S Zamboni G Calandra-Buonaura G Serafini M Lui F et al. Impaired fear processing in right mesial temporal sclerosis: a fMRI study. Brain Res Bull. (2004) 63:269–81. 10.1016/j.brainresbull.2004.03.005 15196652

[14] Bonora A Benuzzi F Monti G Mirandola L Pugnaghi M Nichelli P. Recognition of emotions from faces and voices in medial temporal lobe epilepsy. Epilepsy Behav. (2011) 20:648–54. 10.1016/j.yebeh.2011.01.027 21459049

[15] Fowler HL Baker GA Tipples J Hare DJ Keller S Chadwick DW. Recognition of emotion with temporal lobe epilepsy and asymmetrical amygdala damage. Epilepsy Behav. (2006) 9:164–72. 10.1016/j.yebeh.2006.04.013 16765649

[16] Meletti S Benuzzi F Rubboli G Cantalupo G Stanzani Maserati M. Impaired facial emotion recognition in early-onset right mesial temporal lobe epilepsy. Neurology. (2003) 60:426–31. 10.1212/WNL.60.3.426 12578923

[17] Meletti S Benuzzi F Cantalupo G Rubboli G Tassinari CA Nichelli P. Facial emotion recognition impairment in chronic temporal lobe epilepsy. Epilepsia. (2009) 50:1547–59. 10.1111/j.1528-1167.2008.01978.x 19175397

[18] Monti G Meletti S. Emotion recognition in temporal lobe epilepsy: a systematic review. Neurosci Biobehav Rev. (2015) 55:280–93. 10.1016/j.neubiorev.2015.05.009 25999121

[19] Kemppainen EJ Nissinen J Pitkanen A. Fear conditioning is impaired in systemic kainic acid and amygdala-stimulation models of epilepsy. Epilepsia. (2006) 47:820–9. 10.1111/j.1528-1167.2006.00542.x 16686646

[20] Elsharkawy AE Pannek H Woermann FG Gyimesi C Hartmann S Aengenendt J. Apical temporal lobe resection; “tailored” hippocampus-sparing resection based on presurgical evaluation data. Acta Neurochir. (2011) 153:231–8. 10.1007/s00701-010-0734-2 20640459

[21] Bonelli SB Powell R Yogarajah M Thompson PJ Symms MR Koepp MJ. Preoperative amygdala fMRI in temporal lobe epilepsy. Epilepsia. (2009) 50:217–27. 10.1111/j.1528-1167.2008.01739.x 18717711 PMC2905610

[22] Blumer D Wakhlu S Davies K Hermann B. Psychiatric outcome of temporal lobectomy for epilepsy: incidence and treatment of psychiatric complications. Epilepsia. (1998) 39:478–86. 10.1111/j.1528-1157.1998.tb01409.x 9596199

[23] Ring HA Moriarty J Trimble MR A. prospective study of the early postsurgical psychiatric associations of epilepsy surgery. J Neurol Neurosurg Psychiatry. (1998) 64:601–4. 10.1136/jnnp.64.5.601 9598674 PMC2170068

[24] Schacher M Winkler R Grunwald T Kraemer G Kurthen M Reed V. Mesial temporal lobe epilepsy impairs advanced social cognition. Epilepsia. (2006) 47:2141–6. 10.1111/j.1528-1167.2006.00857.x 17201715

[25] Broicher S Kuchukhidze G Grunwald T Kraemer G Kurthen M Trinka E. Association between structural abnormalities and fMRI response in the amygdala in patients with temporal lobe epilepsy. Seizure. (2010) 19:426–31. 10.1016/j.seizure.2010.06.012 20638303

[26] Schacher M Haemmerle B Woermann FG Okujava M Huber D Grunwald T et al. Amygdala fMRI lateralizes temporal lobe epilepsy. Neurology. (2006) 66:81–7. 10.1212/01.wnl.0000191303.91188.00 16401851

[27] Ashburner J. A fast diffeomorphic image registration algorithm. Neuroimage. (2007) 38:95–113. 10.1016/j.neuroimage.2007.07.007 17761438

[28] Tierney TM Weiss-Croft LJ Centeno M Shamshiri EA Perani S Baldeweg T. FIACH: a biophysical model for automatic retrospective noise control in fMRI. Neuroimage. (2016) 124:1009–20. 10.1016/j.neuroimage.2015.09.034 26416652

[29] Friston K. A theory of cortical responses. Philos Trans R Soc Lond B Biol Sci. (2005) 360:815–36. 10.1098/rstb.2005.1622 15937014 PMC1569488

[30] Pieper S Lorensen B Schroeder W Kikinis R. The NA-MIC kit: ITK, VTK, pipelines, grids and 3D slicer as an open platform for the meidcal image computing community. Proc 3rd IEEE Int Symp Biomed Imaging. (2006) 1:698. 10.1109/ISBI.2006.162501227295638

[31] Frazier JA Chiu S Breeze JL Makris N Lange N Kennedy DN. Structural brain magnetic resonance imaging of limbic and thalamic volumes in pediatric bipolar disorder. Am J Psychiatry. (2005) 162:1256–65. 10.1176/appi.ajp.162.7.1256 15994707

[32] Desikan RS Segonne F Fischl B Quinn BT Dickerson BC Blacker D. An automated labeling system for subdividing the human cerebral cortex on MRI scans into gyral based regions of interest. Neuroimage. (2006) 31:968–80. 10.1016/j.neuroimage.2006.01.021 16530430

[33] Makris N Goldstein JM Kennedy D Hodge SM Caviness VS Faraone SV. Decreased volume of left and total anterior insular lobule in schizophrenia. Schizophr Res. (2006) 83:155–71. 10.1016/j.schres.2005.11.020 16448806

[34] Goldstein JM Seidman LJ Makris N Ahern T O'Brien LM Caviness VS Jr. Hypothalamic abnormalities in schizophrenia: sex effects and genetic vulnerability. Biol Psychiatry. (2007) 61:935–45. 10.1016/j.biopsych.2006.06.027 17046727

[35] Malone IB Leung KK Clegg S Barnes J Whitwell JL Ashburner J. Accurate automatic estimation of total intracranial volume: a nuisance variable with less nuisance. Neuroimage. (2015) 104:366–72. 10.1016/j.neuroimage.2014.09.034 25255942 PMC4265726

[36] Lehrl S Triebig G Fischer B. Multiple choice vocabulary test MWT as a valid and short test to estimate premorbid intelligence. Acta Neurol Scand. (1995) 91:335–45. 10.1111/j.1600-0404.1995.tb07018.x 7639062

[37] Froming K Levy M Schaffer S Ekman P. The comprehensive affect testing system. Psychology Software, Inc. (2006).

[38] Brand JG Mindt MR Schaffer SG Alper KR Devinsky O Barr WB. Emotion processing bias and age of seizure onset among epilepsy patients with depressive symptoms. Epilepsy Behav. (2012) 25:552–7. 10.1016/j.yebeh.2012.09.031 23153721

[39] Ekman P Friesen WV. Pictures of facial affect. Palo Alto, CA: Consulting Psychologist's Press (1976).

[40] Hermann C Buss U Snaith RP. Hospital Anxiety and Depression Scale – German Version (HADS-D). Bern: Huber (1995).

[41] Soeder BM Gleissner U Urbach H. Causes, presentation and outcome of lesional adult onset mediotemporal lobe epilepsy. J Neurol Neurosurg Psychiatry. (2009) 80:894–9. 10.1136/jnnp.2008.165860 19357127

[42] Ekizoglu E Tuzun E Woodhal M Lang B Jacobson L Icoz S. Investigation of neuronal autoantibodies in two different focal epilepsy syndromes. Epilepsia. (2014) 55:414–22. 10.1111/epi.12528 24502404

[43] Bien CG Urbach H Schramm J Soeder BM Becker AJ Voltz R. Limbic encephalitis as a precipitating event in adult-onset temporal lobe epilepsy. Neurology. (2007) 12:1236–44. 10.1212/01.wnl.0000276946.08412.ef 17875912

[44] Wagner J Weber B Elger CE. Early and chronic gray matter volume changes in limbic encephalitis revealed by voxel-based morphometry. Epilepsia. (2015) 56:754–61. 10.1111/epi.12968 25809952

[45] Janszky J Ebner A Kruse B Mertens M Jokeit H Seitz RJ. Functional organization of the brain with malformations of cortical development. Ann Neurol. (2003) 53:759–67. 10.1002/ana.10545 12783422

[46] Kuchukhidze G Siedentopf C Unterberger I Koppelstaetter F Kronbichler M Zamarian L. Language dominance in patients with malformations of cortical development and epilepsy. Front Neurol. (2019) 10:1209. 10.3389/fneur.2019.01209 31824399 PMC6881376

[47] Marusic P Najm IM Ying Z Prayson R Rona S Nair D. Focal cortical dysplasias in eloquent cortex: functional characteristics and correlation with MRI and histopathologic changes. Epilepsia. (2002) 43:27–32. 10.1046/j.1528-1157.2002.00801.x 11879383

[48] Labudda K Mertens M Steinkroeger C Bien CG Woermann FG. Lesion side matters - an fMRI study on the association between neural correlates of watching dynamic fearful faces and their evaluation in patients with temporal lobe epilepsy. Epilepsy Behav. (2014) 31:321–8. 10.1016/j.yebeh.2013.10.014 24210457

[49] Bora E Meletti S. Social cognition in temporal lobe epilepsy: a systematic review and meta-analysis. Epilepsy Behav. (2016) 60:50–7. 10.1016/j.yebeh.2016.04.024 27179192

[50] Toller G Adhimoolam B Grunwald T Huppertz HJ Kurthen M Rankin KP. Right mesial temporal lobe epilepsy impairs empathy-related brain responses to dynamic fearful faces. J Neurol. (2015) 262:729–41. 10.1007/s00415-014-7622-2 25572160

[51] Steiger BK Muller AM Spirig E Toller G Jokeit H. Mesial temporal lobe epilepsy diminishes functional connectivity during emotion perception. Epilepsy Res. (2017) 134:33–40. 10.1016/j.eplepsyres.2017.05.004 28535409

[52] Golouboff N Fiori N Delalande O Fohlen M Dellatolas G Jambaque I. Impaired facial expression recognition in children with temporal lobe epilepsy: impact of early seizure onset on fear recognition. Neuropsychologia. (2008) 46:1415–28. 10.1016/j.neuropsychologia.2007.12.019 18249422

[53] Meletti S Benuzzi F Nichelli P Tassinari CA. Damage to the right hippocampal-amygdala formation during early infancy and recognition of fearful faces: neuropsychological and fMRI evidence in subjects with temporal lobe epilepsy. Ann N Y Acad Sci. (2003) 1000:385–8. 10.1196/annals.1280.036 14766652

[54] Canli T Cooney RE Goldin P Shah M Sivers H Thomason ME. Amygdala reactivity to emotional faces predicts improvement in major depression. Neuroreport. (2005) 16:1267–70. 10.1097/01.wnr.0000174407.09515.cc 16056122

