# Supplementary material for: Emotional Recognition in Patients With Mesial Temporal Epilepsy Associated With Enlarged Amygdala
Source: Front Neurol. 2022 Jan 21;12:803787. doi: 10.3389/fneur.2021.803787 (PMC8815259; doi:10.3389/fneur.2021.803787)
Supplement: Supplementary file 1 [file Table_1.docx]

| Supplementary Table 1. Individually defined peak coordinates in left and right amygdalae | | | | | | | | |
| --- | --- | --- | --- | --- | --- | --- | --- | --- |
|  | right amygdala | | | | left amygdala | | | |
| Subject | x | y | z | voxel | x | y | z | voxel |
| patient 1 | 23.665123 | -2.069444 | -20.455247 | 972 | -24.014184 | -3.457447 | -19.347518 | 846 |
| patient 2 | 23.491391 | -2.483444 | -19.333113 | 755 | -24.204198 | -2.335878 | -20.156489 | 786 |
| patient 3 | 24.105820 | -0.743386 | -20.851852 | 1134 | -24.188997 | -2.177462 | -20.616681 | 1127 |
| patient 4 | 22.839831 | -2.034247 | -18.889884 | 949 | -23.195021 | -3.923237 | -18.491701 | 723 |
| patient 5 | 22.483923 | -2.311897 | -20.374598 | 933 | -23.610253 | -3.480337 | -18.908006 | 712 |
| patient 6 | 23.539726 | -2.545890 | -19.350000 | 730 | -24.800279 | -3.779330 | -19.621508 | 716 |
| patient 7 | 23.010989 | -2.981456 | -20.033654 | 728 | -20.883073 | -4.707127 | -18.006682 | 449 |
| patient 8 | 23.549497 | -2.861577 | -19.799497 | 596 | -23.201183 | -5.356509 | -18.084320 | 338 |
| patient 9 | 23.996047 | -1.264822 | -19.962451 | 759 | -24.657854 | -3.195956 | -18.461897 | 643 |
| patient 10 | 24.160319 | -1.371007 | -19.951474 | 814 | -24.180477 | -3.645289 | -19.607264 | 881 |
| patient 11 | 22.290441 | -2.810294 | -19.352206 | 680 | -23.990400 | -5.066400 | -19.456800 | 625 |
| patient 12 | 24.112258 | -1.387742 | -20.249032 | 775 | -23.410392 | -4.472892 | -18.851657 | 664 |
| patient 13 | 24.112832 | -3.356195 | -17.991150 | 678 | -23 419195 | -5390706 | -19401893 | 581 |
| patient 14 | 23.497159 | -2.640625 | -19.596591 | 1056 | -24.500000 | -3.775424 | -19.730932 | 708 |
| patient 15 | 24.631324 | -1.433800 | -19.165770 | 929 | -24.202790 | -2.443133 | -19.306867 | 932 |
| patient 16 | 23.042994 | -0.990446 | -21.660828 | 942 | -25.107320 | -2.778536 | -19.820099 | 806 |
| patient 17 | 23.693456 | -2.338691 | -19.389782 | 871 | -23.577068 | -5.419173 | -18.166353 | 532 |
| patient 18 | 24.743548 | -1.450000 | -19.927419 | 930 | -23.985203 | -4.350185 | -18.769420 | 811 |
| patient 19 | 24.216626 | -2.084345 | -19.607403 | 824 | -25.165125 | -3.457728 | -19.361295 | 757 |
| patient 20 | 23.816118 | -2.153802 | -19.464245 | 881 | -23.934114 | -5.106149 | -18.722548 | 683 |
| patient 21 | 23.923077 | -3.003846 | -18.534615 | 780 | -23.113716 | -3.840563 | -18.195193 | 853 |
| patient 22 | 23.973363 | -2.096004 | -18.820755 | 901 | -24.185596 | -2.642659 | -19.930748 | 1083 |
| healthy control 1 | 24.035314 | -2.034753 | -19.957399 | 892 | -23.140936 | -1.972112 | -20.775896 | 1004 |
| healthy control 2 | 24.520115 | -2.454023 | -20.129310 | 522 | -22.564388 | -4.693164 | -20.575517 | 629 |
| healthy control 3 | 24.689655 | -1.418719 | -20.160099 | 609 | -22.436842 | -4.998496 | -20.314286 | 665 |
| healthy control 4 | 23.062010 | -1.709530 | -19.917102 | 766 | -23.559390 | -3.991974 | -20.465490 | 623 |
| healthy control 5 | 24.545455 | -2.966468 | -19.978390 | 671 | -22.969064 | -4.750836 | -20.385452 | 598 |
| healthy control 6 | 23.154088 | -2.641509 | -20.515723 | 477 | -23.192699 | -4.856537 | -20.426995 | 589 |
| healthy control 7 | 24.497041 | -4.014793 | -18.458580 | 507 | -23.343121 | -5.501678 | -20.224832 | 596 |
| healthy control 8 | 24.048267 | -3.001856 | -20.092203 | 808 | -22.646523 | -5.024007 | -20.001656 | 604 |
| healthy control 9 | 23.426702 | -3.426702 | -19.073298 | 573 | -23.237850 | -5.083211 | -19.789396 | 679 |
| healthy control 10 | 23.988550 | -3.490076 | -19.477099 | 655 | -23.718750 | -4.181250 | -19.186607 | 560 |
| healthy control 11 | 23.684874 | -3.428571 | -19.968908 | 595 | -24.291045 | -4.194030 | -20.039801 | 603 |
| healthy control 12 | 22.326336 | -2.946565 | -20.162214 | 786 | -23.151099 | -5.175824 | -19.752747 | 546 |
| healthy control 13 | 23.833801 | -2.585554 | -20.049088 | 713 | -21.970728 | -4.673259 | -21.234968 | 632 |
| healthy control 14 | 22.771300 | -3.246637 | -20.062780 | 669 | -23.745690 | -4.877155 | -19.517241 | 696 |
| healthy control 15 | 23.248851 | -2.462481 | -18.813170 | 653 | -23.578475 | -4.921525 | -19.224215 | 669 |
| healthy control 16 | 23.800143 | -1.500000 | -19.972779 | 698 | -22.795638 | -3.971729 | -19.146204 | 619 |
| healthy control 17 | 23.873614 | -2.687361 | -19.776053 | 451 | -23.741453 | -4.489316 | -19.611111 | 702 |
